# Aspergillus Species as a Rare Cause of Primary Mycotic Aneurysm With Aortoenteric Fistula

**DOI:** 10.7759/cureus.75960

**Published:** 2024-12-18

**Authors:** Fahad Alshubaily, Jumana A Fatani, Abdullah Almufarrih, Isam Osman

**Affiliations:** 1 General Surgery, King Saud Medical City, Riyadh, SAU; 2 Surgery, Specialized Medical Center, Riyadh, SAU; 3 Vascular Surgery, King Saud Medical City, Riyadh, SAU

**Keywords:** abdominal aortic aneurysm, aortoenteric, fistula, fungal, gastrointestinal bleeding, infection, primary aortoenteric fistula, secondary aortoenteric fistula

## Abstract

Aortoenteric fistula (AEF) is an abnormal connection between the aorta and the adjacent gastrointestinal (GI) tract and is often misdiagnosed in clinical practice. We present the case of a 65-year-old male, who presented with upper GI bleeding and melena. The patient underwent upper and lower GI examinations with no conclusive findings. A computed tomography scan of the abdomen was suggestive of an AEF. The patient experienced a sudden episode of hematemesis with hemorrhagic shock in the ward, leading to an emergent surgery for bleeding control and repair of the aortic aneurysm and AEF with straight aortic interposition graft and primary repair of the duodenum along with debridement for whitish mycotic debris. A tissue culture from the aortic aneurysm during surgery revealed *Aspergillus* species infection. AEF is a life-threatening condition with high morbidity and mortality rates, often making it difficult to diagnose. Early surgical intervention is crucial to prevent a fatal outcome. Although rare, fungal infection should be considered in a primary AEF.

## Introduction

Aortoenteric fistula (AEF) is an abnormal connection between the aorta and the adjacent gastrointestinal (GI) tract [1,2]. Although rare, it is a life-threatening condition [1,2]. AEF is a rare cause of GI bleeding and can cause sepsis [1,2]. Depending on the cause, AEF can be classified into primary and secondary AEF [1,2]. Primary AEF is a connection between the aorta and the adjacent part of the GI tract [1,2], most commonly caused by compression against an abdominal aortic aneurysm [2]. Secondary AEF develops when there is a connection between an aortic graft and a bowel [2]. When it is a complication of an abdominal aortic aneurysm (AAA) repair [2], it can follow any aortic reconstruction, such as endovascular aneurysm stent-graft repair or the use of bare metal aortic stents [1]. Although fungal infections in vascular grafts are uncommon, immunocompromised individuals have a higher risk of developing opportunistic fungal infections [1].

## Case presentation

**Figure 1:**
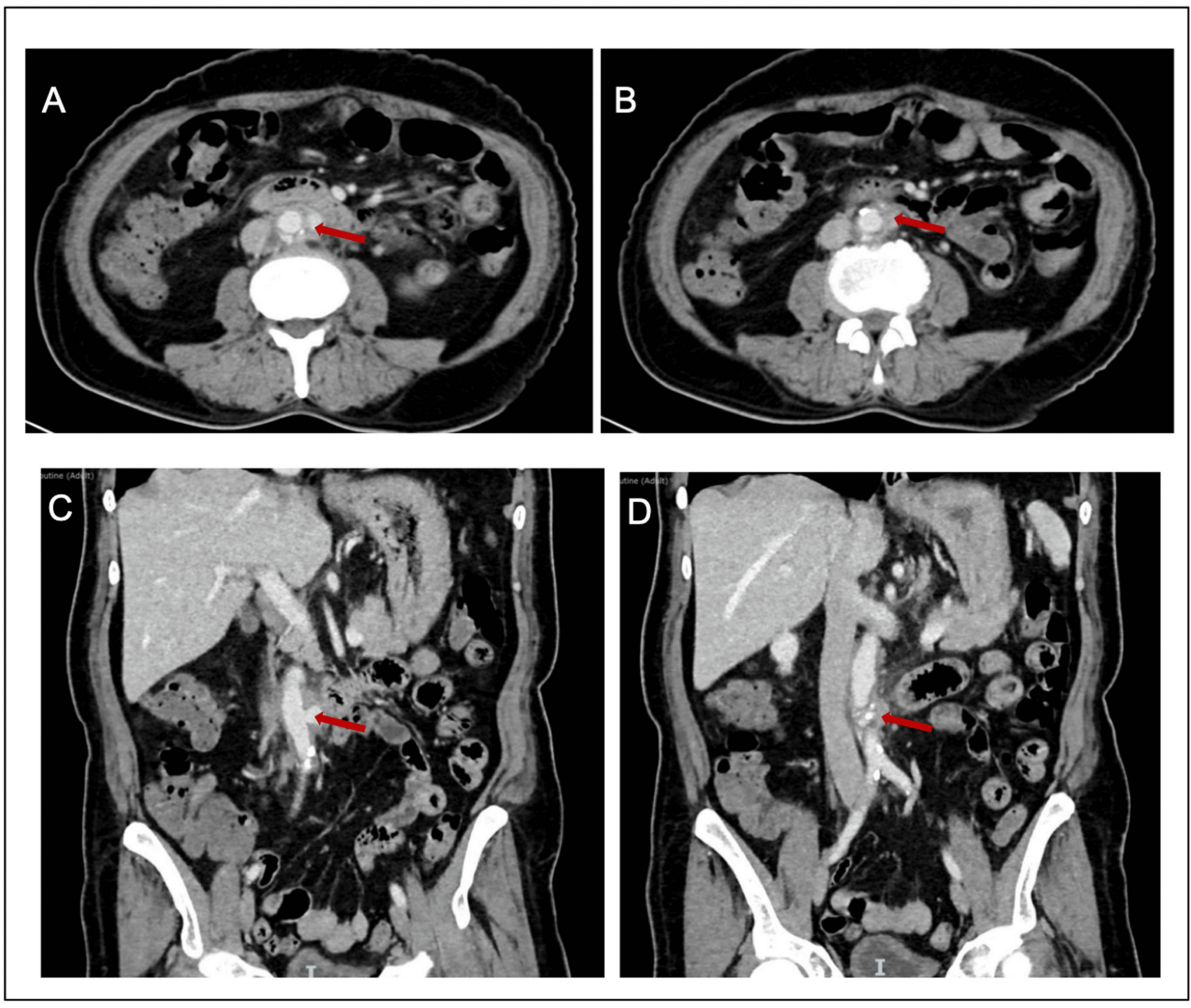
A and B: Contrast-enhanced computed tomography (CT) of the abdomen axial cuts showing infrarenal abdominal aorta pseudoaneurysm with an adjacent hematoma and fat stranding concerning for leakage. C and D: Contrast-enhanced CT of the abdomen coronal cuts showing infrarenal abdominal aorta pseudoaneurysm with an adjacent hematoma and fat stranding.

A 65-year-old male with a known case of hypertension and ischemic heart disease who had undergone right coronary artery stenting one month before presentation was admitted for upper GI bleeding and melena. The patient reported a history of abdominal pain for five days and vomiting blood for three days before presentation that became severe at the time of presentation and was associated with melena. There was no history of abdominal trauma or intervention. He was resuscitated with blood and fresh frozen plasma and was started on omeprazole infusion. The patient underwent upper and lower GI scopes. Esophagogastroduodenoscopy (EGD) showed a normal esophagus with no evidence of bleeding in the stomach or duodenal bulb, and the first and second portions of the duodenum were normal. The entire colon and ileum examined on the colonoscopy were normal. A computed tomography (CT) scan of the abdomen showed focal saccular left lateral outpouching of the infrarenal descending abdominal aorta, measuring 1.0 × 0.9 cm. The neck measured about 0.5 cm which suggested an infrarenal abdominal aorta pseudoaneurysm. There was surrounding circumferential fat stranding and hematoma concerning for leakage (Figure 1).

**Figure 2:**
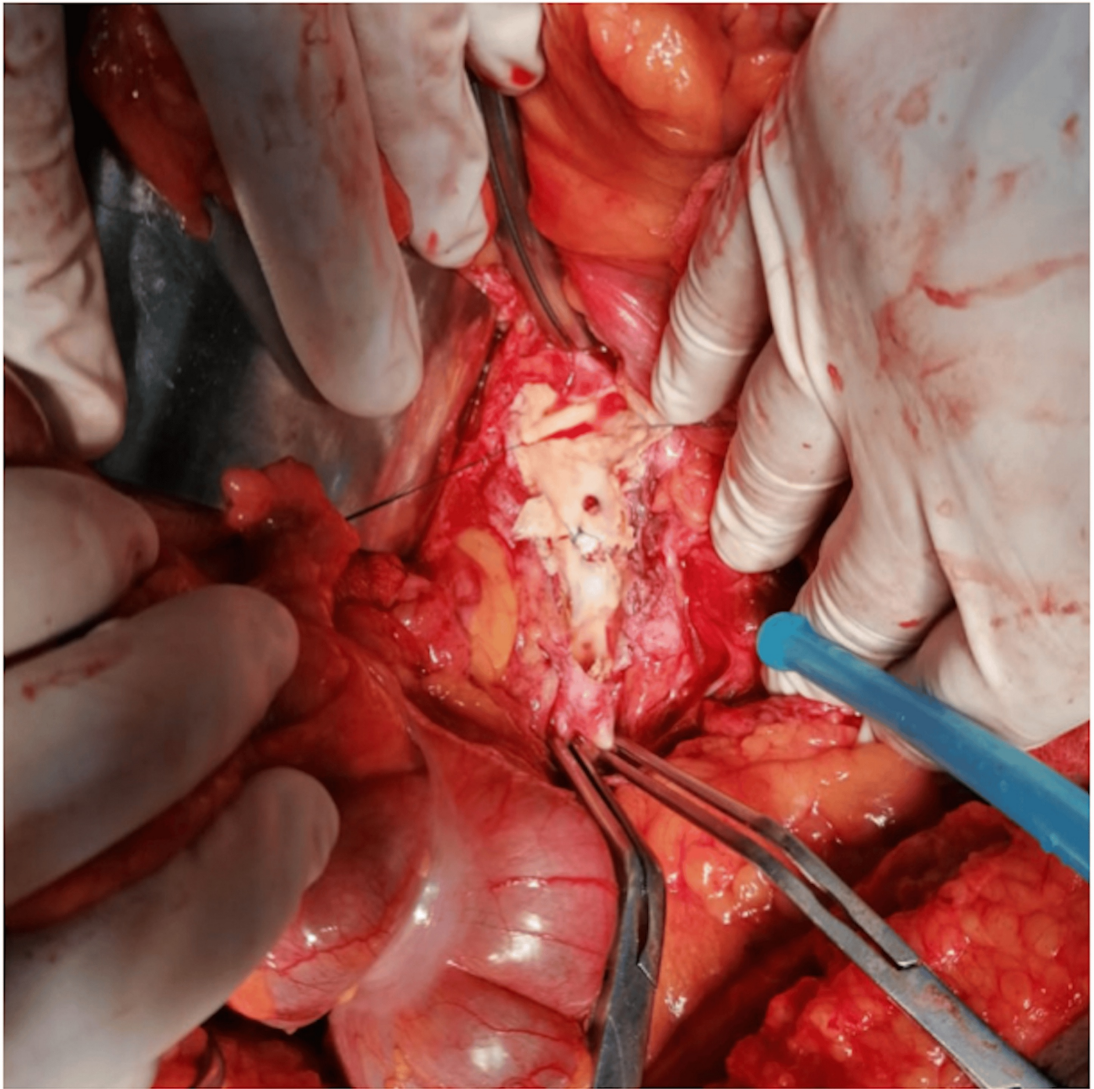
Whitish mycotic aortic aneurysm.

**Figure 3:**
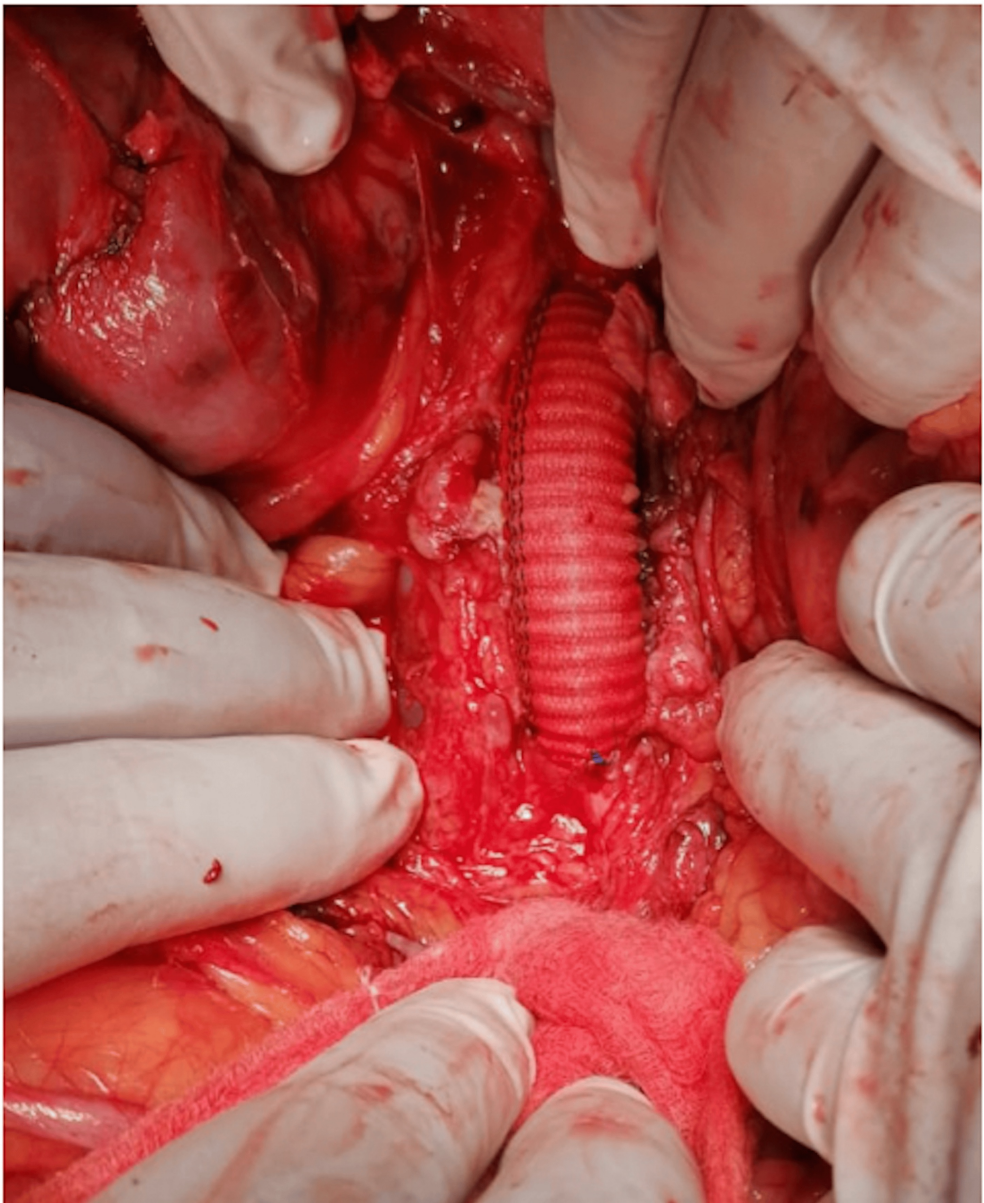
The aneurysm after aortic interposition graft repair.

On the third day, he suffered from severe hematemesis with hemorrhagic shock. Hence, he was immediately shifted to the operating room. Intraoperative finding of a fistula between the aorta and the fourth part of the duodenum was discovered with whitish dense fibrotic debris noted inside the aneurysm and in the fistula tract as well as retroperitoneum with clots and active bleeding in the duodenum (Figures 2, 3). He underwent repair of the AEF with a straight aortic interposition graft, along with primary repair of the duodenum and debridement of retroperitoneal whitish debris.

Following the surgery, the patient was monitored in the intensive care unit and later transferred to a regular ward once stabilized. A tissue culture from the aortic aneurysm during surgery revealed an Aspergillus species infection, prompting the patient to receive antifungal treatment. The patient was started on voriconazole and augmentin and blood cultures were taken which returned negative. The patient was discharged in good condition. On the clinic follow-up, the patient was fine with no complications.

## Discussion

The incidence of primary AEFs has been described as 0.04-0.07% among patients who passed away due to a massive GI hemorrhage, with a greater incidence in AAA patients, accounting for 0.69-2.36% [2]. A range of 0.36-1.6% has been reported for secondary AEF [2]. AEF is more prevalent in males. The male-to-female ratio in primary AEF is 3:1 and 8:1 for secondary AEF [2]. The risk factors for developing AEFs include atherosclerosis, previous surgical history of gastric or aortic reconstruction, an outcome of malignant tumors, duodenal ulcers, foreign materials, and complications after radiotherapy and infection, including syphilis, tuberculosis, and bacterial or fungal aortic infection [2,3]. Aortitis, an inflammation within the aneurysmal wall, has been proposed as a factor that could lead to cell degradation, pressure necrosis, or mycotic erosion, ultimately resulting in fistula formation [3,4]. Inflammatory aneurysms account for 3-10% of AAAs [4]. Infectious aortitis is an uncommon but fatal condition that must be differentiated from other inflammatory conditions [4]. Bacteria typically enter the aortic wall through the vasa vasorum, often associated with an underlying pathology [4]. This can lead to the development of an aneurysm or pseudoaneurysm that erodes into nearby structures [4]. *Salmonella* or *Klebsiella* are commonly reported as the causative pathogens in primary mycotic aneurysms. However, there have been case reports of unusual pathogenic organisms, including *Coxiella burnetii*, which is linked to Q-fever [5].

The most common type of AEF is the gastroduodenal fistula, and the third and fourth sections of the duodenum are the most frequently affected areas [1], particularly the third part, which is close to the aorta [2].

AEF is usually difficult to diagnose and often misdiagnosed due to the non-specific presentation [2]. Although the classical triad to diagnose AEF includes GI bleeding, pain, and a pulsating mass, it is not frequently seen and is appreciated in only 11% of cases [2,4]. The upper GI bleeding can range from mild to severe hemorrhage [1]. The most common initial presentations are bleeding and sepsis [2]. Other symptoms include unexplained abdominal pain, fever, or sepsis [1]. Although the cause of AEF is not fully understood, it is suggested that the formation of a fistula is the result of a combination of a chronic low-grade infection of the aortic graft and repeated pressure on the bowel caused by the pulsations of the aorta [1].

If there is suspicion of AEF, the initial diagnostic approach involves a CT scan, followed by endoscopy and arteriography [1]. CT angiography (CTA) is capable of identifying a fistula in 35% of cases, while EGD can detect fistulas in 25% of cases [2]. Exploratory laparotomies show the highest sensitivity and specificity, ranging from 91% to 100% [2]. Laparotomy is recommended for GI bleeding with an unclear source [3]. CT scans help locate AEFs and identify infections or abscesses in the abdomen [2]. CTA can assess the size and location of an aneurysm, but the detection of active contrast extravasation into the bowel is only positive in 26% of cases [3]. CT findings of primary AEF include ectopic gas in the aneurysmal sac and enteric contrast in the sac. The absence of a fat plane between the vessel and bowel lumen may indicate the early development of an AEF [6]. Further observations for secondary AEF involve inflammation of soft tissue around the graft and thickening of the nearby bowel wall [6]. Endoscopy is the most successful approach for determining the source of GI bleeding. Most AEFs are located in the third or fourth part of the duodenum, frequently overlooked during gastroscopy, which typically assesses only the second segment [2].

The primary goal in managing AEF is to control bleeding, treat infection, and ensure adequate distal blood flow [1]. This involves aggressive fluid and blood transfusions with invasive monitoring for resuscitation [1]. The timing and type of surgical repair depend on various factors, such as the severity of clinical presentation, patient comorbidities, and the type of AEF [4]. Open surgical repair typically includes aortic vascular control, debridement of infected and necrotic tissue, repair or resection of the intestinal defect, and in situ aortic reconstruction with a prosthetic graft [4]. Meanwhile, revascularization is usually performed immediately in primary AEF [4]. Managing secondary AEF involves removing the graft and creating an extra-anatomic bypass. Endovascular repair is a minimally invasive alternative for patients deemed unsuitable for surgery, especially for rapid bleeding control [4]. It can also serve as a temporary measure before open repair for patients with sepsis or other infectious complications or as an adjunct to palliative treatment [1,3,4]. Treatment of infected aneurysms mainly involves a combination of antibiotic therapy, surgical debridement, and revascularization if feasible [4]. Broad-spectrum antibiotic therapy should begin once the diagnosis is confirmed, covering Gram-positive, Gram-negative, and anaerobic bacteria, and should continue postoperatively based on culture reports [1]. The ideal duration of antibiotic therapy is uncertain and varies depending on several factors, with six weeks of antibacterial therapy generally recommended [4].

Although rare, some cases have reported fungal infection in vascular graft infection [1]. However, due to advancements in medical science that have made immunocompromised patients eligible for high-risk vascular procedures, it may be more common than previously thought, thereby increasing the likelihood of opportunistic fungal infections, with *Candida* being one of the most common organisms found in aortic graft infections [1].

A case of primary AEF was reported and treated with endovascular aneurysm repair followed by additional surgical procedures, including an extra-anatomic bypass, aortic ligation, duodenal resection with primary anastomosis, and placement of a gastrojejunostomy tube. The cultures from the surgery showed the presence of *Candida albicans* [5]. To our knowledge, our case represents the first reported case of primary mycotic AEF caused by a fungal infection, specifically Aspergillus species. The untreated mortality rate of AEF can be as high as 100%, and sepsis remains a life-threatening complication, with failure to control it leading to mortality rates of 60% [2]. Therefore, adding empiric antifungal therapy in patients suspected of having an aortoduodenal fistula may be reasonable [1].

## Conclusions

AEF is a life-threatening condition with high morbidity and mortality rates, often making it difficult to diagnose. Early surgical intervention is crucial to prevent a fatal outcome, especially when a patient experiences severe GI bleeding. Although rare, fungal infection should be considered in a primary AEF. Immunocompromised patients undergoing risky vascular procedures are at an increased risk of opportunistic fungal infections, with *Candida* being one of the most common organisms found in aortic graft infections.
